# Anisotropy in Additively Manufactured Concrete Specimens under Compressive Loading—Quantification of the Effects of Layer Height and Fiber Reinforcement

**DOI:** 10.3390/ma16155488

**Published:** 2023-08-06

**Authors:** Sahil Surehali, Avinaya Tripathi, Narayanan Neithalath

**Affiliations:** School of Sustainable Engineering and Built Environment, Arizona State University, Tempe, AZ 85287, USA; ssurehal@asu.edu (S.S.); atripa22@asu.edu (A.T.)

**Keywords:** 3D printing, anisotropy, interfaces, compression, interface-parallel cracking, fiber reinforcement

## Abstract

This paper analyzes the effect of print layer heights and loading direction on the compressive response of plain and fiber-reinforced (steel or basalt fiber) 3D printed concrete. Slabs with three different layer heights (6, 13, and 20 mm) are printed, and extracted cubes are subjected to compression (i) along the direction of printing, (ii) along the direction of layer build-up, and (iii) perpendicular to the above two directions. Digital image correlation (DIC) is used as a non-contact means to acquire the strain profiles. While the 3D printed specimens show lower strengths, as compared to cast specimens, when tested in all three directions, this effect can be reduced through the use of fiber reinforcement. Peak stress and peak strain-based anisotropy coefficients, which are linearly related, are used to characterize and quantify the directional dependence of peak stress and strain. Interface-parallel cracking is found to be the major failure mechanism, and anisotropy coefficients increase with an increase in layer height, which is attributable to the increasing significance of interfacial defects. Thus, orienting the weaker interfaces appropriately, through changes in printing direction, or strengthening them through material modifications (such as fiber reinforcement) or process changes (lower layer height, enables attainment of near-isotropy in 3D printed concrete elements.

## 1. Introduction

The process of 3D concrete printing (3DCP), also referred to as digital fabrication technique or digital concrete, is gaining significant attention among researchers and practitioners globally [1,2,3]. As a digital fabrication technique, it aims to reform the traditionally conservative and innovation-resistant construction industry into a modern, digitized industry with unprecedented design and construction freedom [4]. The liberty of design optimization offered by 3DCP allows for the use of materials only where it is structurally or functionally needed, thereby reducing the overall material usage and allowing levels of materials-related sustainability previously unheard of in construction [5]. Furthermore, the elimination of formwork, which represents 35–60% of the overall cost of concrete structures and consumes significant time for its fabrication and erection, results in cost savings and productivity enhancements in the construction sector [6]. It has also been reported that 3DCP technology will potentially create high-end technology-based jobs in the construction industry while also enhancing field safety conditions by eliminating dangerous activities (such as working at heights on scaffolding) [7,8,9].

Different 3DCP technologies have been developed over the past few years. These technologies are, mainly, either powder bed-based or extrusion-based. The powder bed-based technique is analogous to the ‘ink-on paper’ printing process [10,11], and it is suitable for manufacturing building components with complex geometries, which can be later assembled onsite [7]. A major advantage of this technique is that inclined structures, overhangs, and arches with great details can be realized without using supporting materials [12,13,14]. However, the dimension of powder bed limits the application of this technique in the construction industry. In extrusion-based printing, cementitious filaments are deposited, layer-by-layer, onto one another until the desired geometry is realized [15,16,17]. From a materials standpoint, extrusion printing has been successfully used along with conventional mortars and concrete, geopolymers or alkali activated binders, polymeric binders, lightweight foam concrete, and fiber-reinforced concrete [18,19,20,21,22,23,24]. A wide array of tests to determine the rheological and fresh state properties, in order to ensure appropriate pumpability, extrudability, and buildability, have been proposed [25,26,27,28] since those characteristics are critical in ensuring the successful implementation of 3DCP. Moreover, they influence the later-age properties of the printed structure as well.

Among the various non-materials related factors that contribute to variation in the mechanical properties of 3D printed concrete elements, printing direction is a frequently mentioned one. This necessitates quantification of the strength parameters in directions along and perpendicular to the direction of printing [29,30]. While some studies report the compressive strength to be generally higher when tested perpendicularly to the printing direction [16,31,32,33], there are others that report otherwise [14,23,34,35,36]. A closer examination reveals that this is mostly due to a confluence of effects that influence the inter-layer or inter-filament bond strength. Reduced cohesion between the printed concrete filaments is attributed to several reasons, including air entrapment during filament deposition, water migration between filaments, and evaporation of surface moisture during the printing process, especially when the layer surfaces are exposed for longer durations before the overlying layer is printed [35,37,38,39]. The absence of vibration before or after the deposition of filaments also increases the interfacial disparities, e.g., inter-layer void sizes, shapes, and fractions, between 3D printed and conventionally cast concrete. Cold joints produced between the filaments, as well as between the layers, further contribute to reducing the compressive, flexural, and tensile strengths of 3D printed concrete specimens compared to the homogeneous, conventionally cast specimens [29,37,39]. Print parameters, such as layer dimensions, print velocity and extrusion rate, nozzle shape and dimensions, as well as nozzle offset distance, also contribute to the inter-layer and inter-filament geometry and properties, hence influencing the directional properties of 3D printed concrete specimens under mechanical loading [29,33,40,41,42].

While a number of past studies have dealt with compressive strength of 3D printed elements, the conclusions on directional dependence are inconsistent—key studies and their findings are shown in Table 1. A majority of these studies acknowledge the presence of anisotropy in the compressive response, but they generally only consider either the inter-layer or the inter-filament interface through nozzle and specimen size control [29,43,44,45], thereby limiting the observations on the effects of the interplay of these interfaces on the properties when tested in different directions. The focus of this work is, therefore, to evaluate the (potential) anisotropic behavior of 3D printed concrete specimens under compression (i.e., when a cube is loaded in either of the three orthogonal directions) as a function of layer height, while incorporating multiple interface types in the same specimens, to simulate realistic conditions. Other process parameters, such as print speed, nozzle shape and diameter, nozzle offset distance, layer width, etc., are kept constant.

Studies in the past have also reported on the influence of fiber reinforcement on the mechanical properties of 3D printed mixtures [23,30,36,44,46]. However, when fibers are incorporated into the mixture, and since they likely align in the direction of printing, anisotropic effects could also manifest. Even though it is more likely that the effects of fibers (at volume fractions generally used in normal performance concretes) that contribute to anisotropy are more discernable under flexural and tensile loads, performance under compression is the focus of this work. Although using fibers longer than the nozzle size is recommended to ensure fiber alignment [36], this paper specifically explores the effect of steel and basalt fibers that are smaller than the nozzle diameter. A mixture that has shown excellent performance in terms of extrudability and buildability, as described in several of our earlier works [47,48], is used here. The results from this study are expected to shed light on the directional dependence of the compressive behavior of plain and fiber-reinforced 3D printed mortars, which is crucial in ensuring that (i) the printed structures can sufficiently resist stresses in multiple directions and (ii) the failure mechanisms and modes are predictable. The results can also be used to inform direction-dependent stresses for design applications, as well as the use of anisotropic/orthotropic design criteria for critical 3D printed structures, if required.

**Table 1:** Salient inferences from past studies, dealing with the directional dependence of compressive strength for 3D printed concretes.

Source	Nozzle Dimensions	Layer Height	Layer Width	Specimen Size	Key Inferences
Liu et al. [31]	30 mm dia.	15 mm	31.5 mm	100 × 100 × 100 mm	Compressive strength (CS) highest in the D3 * direction while it was similar in the D1 * and D2 * directions.
Ye et al. [43]	10 × 50 mm	10 mm	50 mm	50 × 50 × 100 mm	Significant anisotropy reported in CS results and it varied with the fiber content. D3 direction had lowest CS.
Xiao et al. [32]	--	10 mm	50 mm	100 × 100 × 100 mm	CS highest in D2 direction and lowest in D3 direction.
Zahabizadeh et al. [49]	60 × 12 mm	10 mm	50 mm	50 × 50 × 100 mm	No anisotropy observed in CS results.
Arunothayan et al. [23]	30 × 15 mm	15 mm	30 mm	25 × 25 × 25 mm	Highest CS reported in the D1 direction for plain and fiber-reinforced mortars, followed by D2 and D3 directions.
Ding et al. [33]	30 mm dia.	15 mm	30 mm	70.7 × 70.7 × 70.7 mm	CS highest in D2 direction, and lowest in D3 direction.
Ma et al. [36]	12 mm dia.	5 mm	20 mm	50 × 50 × 50 mm	CS similar in D1 and D3 directions and lower in the D2 direction.
Rahul et al. [34]	30 × 30 mm	30 mm	30 mm	50 × 50 × 50 mm	No anisotropy observed in CS, but CS lower by 12–22% compared to cast specimens.
Wolfs et al. [29]	--	9.5 mm	50 mm	40 × 40 × 40 mm	No anisotropy observed.
Zhang et al. [50]	20 mm dia.	12–15 mm	22–24 mm	100 × 100 × 100 mm	Highest CS reported in D2 direction, followed by D3 and D1 directions.
Nematollahi et al. [45]	25 × 15 mm	15 mm	25 mm	50 × 25 × 30 mm	(i) No anisotropy in CS of plain geopolymer mortars. (ii) For fiber-reinforced geopolymer mortars, highest CS in the D2 direction followed by D1 and D3 directions.
Sanjayan et al. [35]	25 × 15 mm	15 mm	25 mm	50 × 25 × 30 mm	Highest and lowest CS in D1 and D3 directions, respectively.
Panda et al. [44]	40 × 10 mm	40 mm	40 mm	40 × 40 × 40 mm	Highest CS in D1 direction followed by D2 and D3 directions, for chopped glass fiber-reinforced samples.
Feng et al. [14]	9 mm dia.	0.0875 mm		70.7 × 70.7 × 70.7 mm	Specimens loaded in D1 direction showed highest CS.
				50 × 50 × 50 mm	

* Direction-1 (D1) is defined along the direction of printing, direction-2 (D2) represents the layer-build-up direction, and the direction perpendicular to both D1 and D2 is denoted as direction-3 (D3).

## 2. Experimental Program

### 2.1. Materials

**Figure 1:**
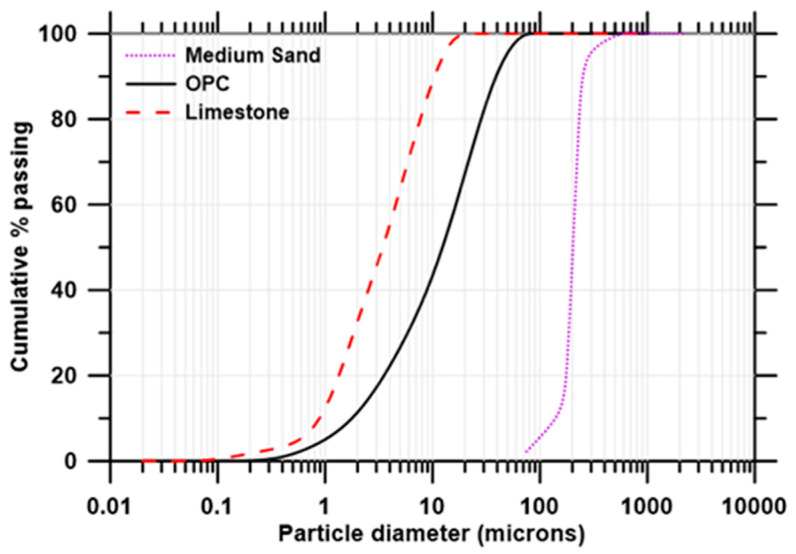
Particle size distributions of the mortar constituents.

**Figure 2:**
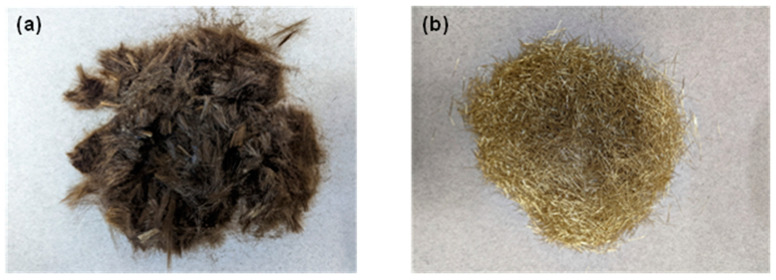
Fibers used in the study: (**a**) chopped Basalt Fiber (BF) and (**b**) chopped Steel Fiber (SF).

Type I Ordinary Portland Cement (OPC), conforming to ASTM C 150 [51], was chosen as the primary cementitious material in the study. Fine limestone (L) powder, conforming to ASTM C 568 [52], was used as a partial replacement of cement (30% by mass). All the mixtures comprised 50% sand, by mass, of the total solids. The physical and chemical properties of the mortar constituents are detailed in Table 2, and their particle size distributions (PSD) are shown in Figure 1. Chopped basalt fibers (BF) and chopped steel fibers (SF), used as fiber reinforcements in this study, are shown in Figure 2, and their physical and mechanical properties are summarized in Table 3. The mixture proportions of plain and fiber-reinforced mortars, shown in Table 4, were derived to ensure adequate extrudability and buildability, as described in our earlier studies [48,53]. A polycarboxylate ether-based superplasticizer (0.35% by mass of the binder) was used in all the mixtures.

### 2.2. 3D Printing of Mortars

**Figure 3:**
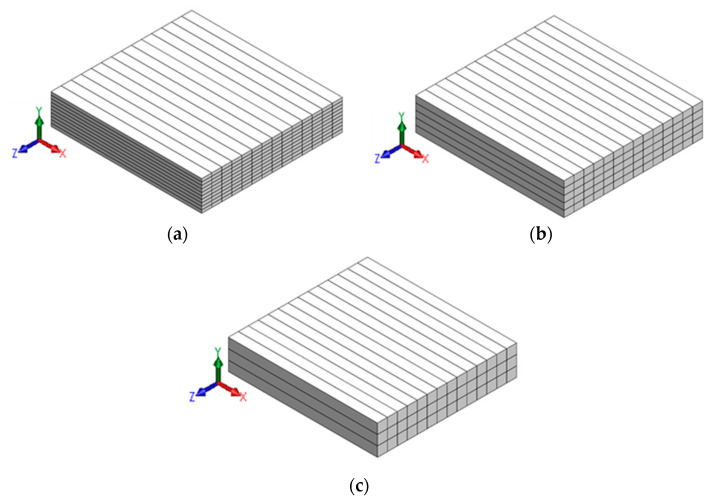
Representative three-dimensional models of mortar slabs (300 × 280 × 60 mm) printed using layer heights of: (**a**) 6 mm, (**b**) 13 mm, and (**c**) 20 mm.

The mortars shown in Table 4 were printed using a gantry 3D printer equipped with a screw extrusion system [53,54]. To obtain a consistent filament cross-section, a circular nozzle of 20 mm diameter was used to print the filaments of a specified layer height (LH) and a fixed layer width (LW) of 20 mm. The print layer heights selected for the study were 6, 13, and 20 mm. To minimize print defects, layer heights smaller or equal to nozzle diameter were selected in this study. It has been reported that the specimens printed using layer heights larger than the nozzle diameter result in larger voids because of the absence of a vertical pressing force and improper filament overlap in the plane of printing [54]. Mortar slabs of dimensions 300 × 280 × 60 mm were printed at a constant in-plane print speed of 50 mm/s. The stepper for the screw extruder was calibrated for a flow rate of about 6, 13, and 20 mL/s to obtain uniform filaments of layer heights 6, 13, and 20 mm, respectively. Figure 3 shows the representative 3D models of printed mortar slabs, of size 300 × 280 × 60 mm, with different layer heights. The 3D printed mortar mixtures were moist-cured at 23 ± 2 °C and >98% relative humidity for 28 d, after which the test specimens were extracted from the slabs by cutting with a diamond-tipped saw.

### 2.3. Compression Testing

**Figure 4:**
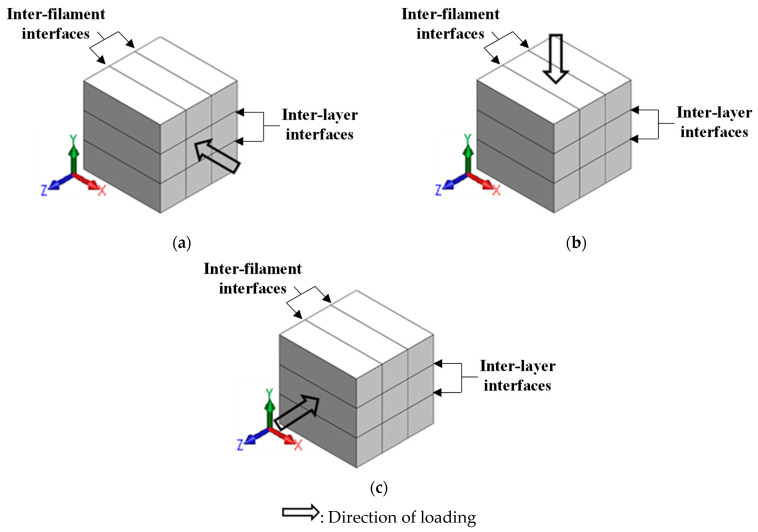
Cubes compressed along: (**a**) direction-1, (**b**) direction-2, and (**c**) direction-3. The representation corresponds to a specimen with a layer height of 20 mm.

Cubes of 60 mm × 60 mm × 60 mm were extracted from the printed mortar slabs. The cubes consisted of 10 layers for a layer height of 6 mm, 4.6 layers for a layer height of 13 mm, and 3 layers for a layer height 20 mm, thereby resulting in significantly different numbers and qualities of layer interfaces. The cubes were subjected to uniaxial compression using a servo-controlled universal testing machine (SBEL CT-110-S) with a capacity of 489 kN. Compression tests were carried out in all three directions (on at least 3 specimens for each direction) for the selected layer heights and mixtures. In this study, direction-1 (D1) is defined as the one along the direction of printing, direction-2 (D2) represents the layer-buildup direction, and the direction perpendicular to both D1 and D2 is denoted as direction-3 (D3). Figure 4 shows the direction of loading for a cube specimen with a layer height of 20 mm. Loading was carried out at a stroke displacement rate of 0.15 mm/min, corresponding to a strain rate of 0.25%/min. Companion mold-cast specimens, of the same composition described earlier, were also tested.

### 2.4. Digital Image Correlation (DIC) Analysis

**Figure 5:**
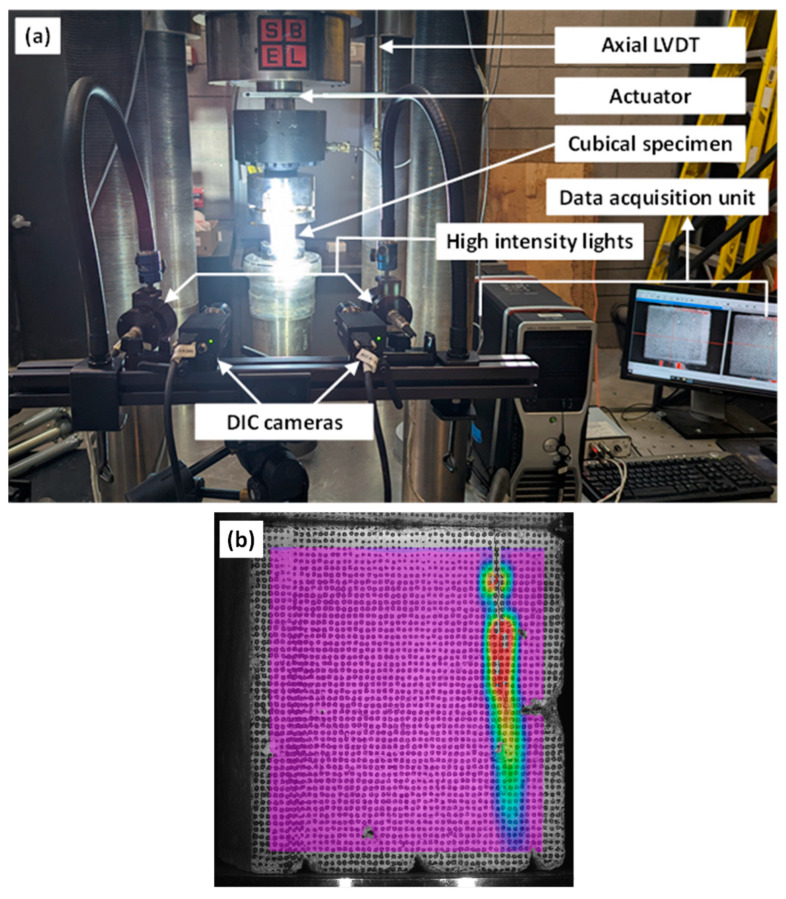
The (**a**) DIC setup and (**b**) a typical DIC strain field (corresponding to cracking) after post-processing the images captured during compression test.

Digital image correlation (DIC) was used as a non-contact means to acquire the strain and displacement field distributions in at least three of the tested cube specimens. The DIC setup shown in Figure 5a comprised two cameras (Point Grey Grasshopper 3 cameras) that focused on the speckled area of interest on the cube illuminated by high-intensity flood lights. The aperture and focus of both cameras were adjusted so that the speckle pattern could be tracked across the images taken during the testing. The cameras were controlled by a data acquisition unit (DAQ), and the frequency of image capture was fixed at 1 Hz. Image analysis for DIC was carried out using VIC-3D software™ from Correlated Solutions (Columbia, SC, USA). The strain resolution of the DIC equipment is about 0.01%, as indicated by the manufacturer. Figure 5b shows a typical DIC strain field after post-processing the images. The process of obtaining Lagrangian strain fields from the displacement field captured by DIC is described elsewhere [55].

## 3. Results and Discussions

### 3.1. Compressive Strength of Mold-Cast Specimens

**Figure 6:**
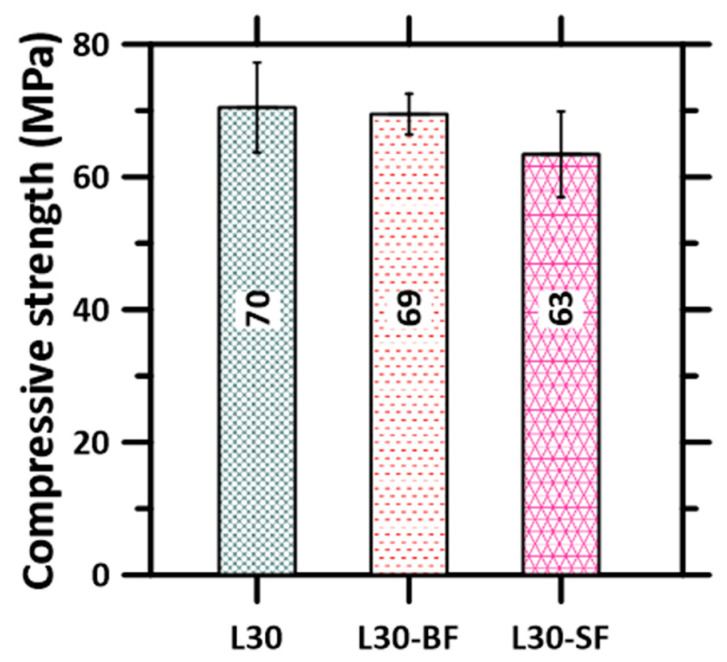
Average compressive strengths and standard deviations determined from 3 to 6 mold-cast cube specimens of plain and fiber-reinforced mortars.

The average 28 day compressive strengths of the mold-casted specimens of plain and fiber-reinforced mixtures are shown in Figure 6. The basalt fiber-reinforced specimens demonstrate comparable strength to that of the unreinforced specimens, attributed to the smaller size of the basalt fibers, and its pore-filling nature is elucidated elsewhere [56,57,58,59]. On the other hand, the compressive strength of the steel fiber-reinforced specimens are, on average, around 10% lower than those of their unreinforced counterparts, due to the formation of voids aided by the presence of stiff, unidirectional steel fibers that impede the packing of particles in the mixture (loosening effect [60]). Note that excessive vibration of the mold-cast specimens was avoided to prevent the settling of steel fibers at the bottom of the mold. Another reason for the lower compressive strength of the steel fiber-reinforced specimen could be that the volume fraction of the steel fibers used in this study is lower than the recommended volume dosage of 2–5% that is needed to enhance performance under compressive loads [61,62] (though a smaller volume fraction of fibers is enough to demonstrate enhanced flexural properties). This also highlights the importance of identifying and utilizing the optimal fiber type and content in the mixture based on the desired properties. However, in this study, we have chosen the fiber content so as to result in toughness enhancement under flexure (as shown in our previous work [54]), which is the main function of stiff fiber reinforcement. Adequate extrudability and buildability have been ensured with this mixture [47,48,63,64], which was used to 3D print the slabs used for further investigations.

### 3.2. Directional and Layer Height Dependence of Compressive Strength of 3D Printed Specimens

**Figure 7:**
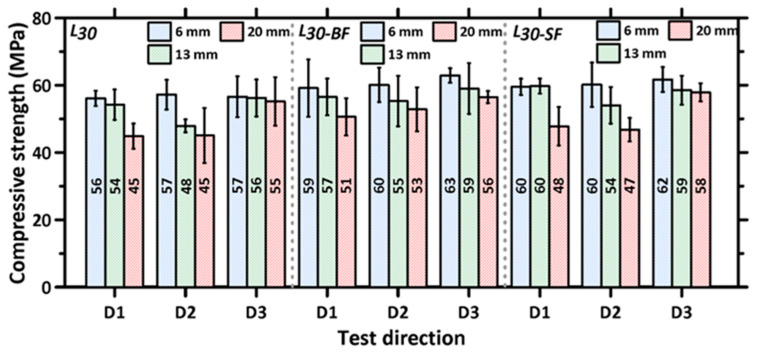
The average compressive strengths and standard deviations determined from 3 to 6 3D printed specimens for L30, L30-BF, and L30-SF mixtures as a function of test direction for each layer height used in the study.

Figure 7 shows the average compressive strengths of the 3D printed specimens as a function of printing direction for each layer height and mixture composition. The compressive strengths of the cubes extracted from the 3D printed slabs, for each layer height and direction of testing, are lower than those of the mold-cast specimens, which is in line with the results reported earlier [30,31,49,65]. This is attributable to the combination of a lack of compaction and layering-induced inhomogeneity. The presence of macropores/voids at the interface of two layers, as has been shown in several previous studies, is the main reason for the lower interfacial bond strength that results in overall lower compressive strengths and demonstrated anisotropy (as will be quantified later) in the mechanical response [66]. Several approaches to enhance inter-layer bond properties—selecting suitable printing parameters, such as minimizing the time interval between the laying of two subsequent layers, nozzle standoff distances, nozzle types, and print velocity—have been reported [29,41,42,67]. Simple alterations to mixture design and processing, such as introducing a small volume of metallic or mineral fibers or changing the layer heights, have been shown to minimize strength loss, as can be seen by comparing Figure 6 and Figure 7. While the compressive strengths of unreinforced specimens are about 20–35% lower than those of the mold-cast specimens (depending on layer heights), the strength loss is restricted to 2–25% in the case of fiber-reinforced mixtures, as noticed from a comparison of Figure 6 and Figure 7. Some of the interfacial effects that lead to strength loss are compensated by the load carrying capacity of the fibers, as well as some inter-layer strength enhancements provided by fibers that do not completely align in the print direction. In the case of basalt fiber, its contribution to cement hydration also likely helps in enhancing mechanical properties [36].

**Figure 8:**
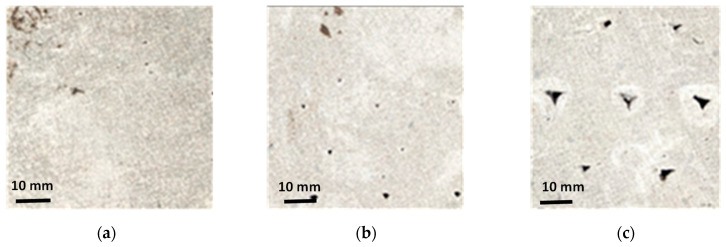
Representative optical images showing voids formed at the inter-filament interfaces for layer heights of: (**a**) 6 mm, (**b**) 13 mm, and (**c**) 20 mm. Cross-sections along the D1 direction are shown in the plane of the images (adopted from author’s previous work [54]).

Figure 7 shows that the compressive strengths are similar for the specimens printed with a layer height of 6 mm, for both unreinforced and fiber-reinforced mixtures, irrespective of the direction of testing. This is attributed to the stronger interfacial (both inter-layer and inter-filament) bonds achieved when smaller layer heights are used. The pressure applied by the print head to achieve smaller layer heights (when the layer height used is smaller than the nozzle diameter) results in stronger inter-layer bonding [54,68]. The interfacial defects formed between the adjacent filaments of two layers (observed when a circular nozzle (A circular nozzle was used in this study as it has been shown that a circular nozzle provides more freedom and ease to change the nozzle angle for the print path [1,69], ensuring consistent filament cross-sections even for the case of non-orthogonal print paths.) is used) are also largely avoided when smaller layer heights are used. Figure 8 shows the cross sections of 3D printed specimens cut perpendicular to the D1 direction for specimens with print layer heights of 6, 13, and 20 mm. It can be seen that, as the layer heights increase, inter-filament voids present in the specimen increase in size. The inter-filament voids are generally absent for the specimens printed with a 6 mm layer height.

**Figure 9:**
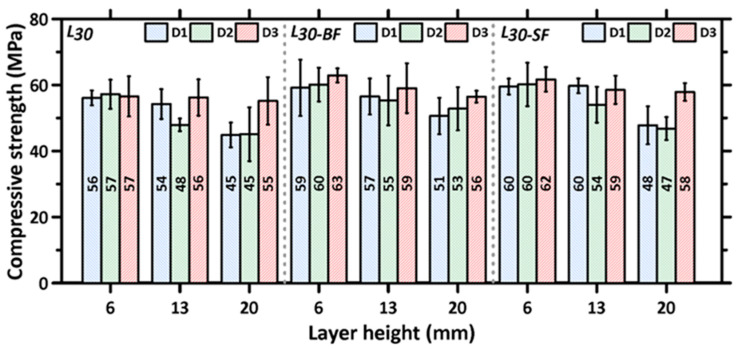
Average compressive strengths and standard deviations from 3 to 6 3D printed specimens for L30, L30-BF, and L30-SF mixtures, as a function of layer height, for each test direction used in the study.

The average compressive strengths of the tested 3D printed specimens, as a function of print layer height, are shown in Figure 9 for both plain and fiber-reinforced mortars used in the study to better elucidate the layer height effects in each direction. It is known that the inter-layer and inter-filament interfaces in 3D printed specimens contribute to strength reduction, which is attributable to the presence of voids and, hence, insufficient contact area for load transfer [43,49,70]. However, the interfacial (inter-layer or inter-filament) bond strength parallel to the stress direction is more significant in compression, as it is amenable to easier separation [36], i.e., interface separation or interface-parallel cracking. In general, therefore, the interfaces perpendicular to stress direction, i.e., the inter-layer and inter-filament interfaces in D2 and D3 directions, respectively (see Figure 4), can be considered to exert lesser influence on the compressive strength of the printed specimens. The compressive strength results for all three mortar mixtures and layer heights used in this study show that the strength is relatively invariant of the print layer height used when the load was applied in the D3 direction. Note that, in the D3 direction, the stress path is perpendicular to the inter-filament interface (see Figure 4). The inter-layer interfaces are, therefore, expected to dominate the failure in the case. With increasing layer height, while the number of inter-layer interfaces decreases, the quality of interfaces also diminishes (N.B.: our previous work has shown that a smaller layer height results in a less porous interface due to the higher nozzle pressure required to achieve smaller layer thickness [54]). The higher number of denser interfaces in a smaller layer height specimen compensates for the effect of a smaller number of more porous interfaces in higher layer height specimens, resulting in the compressive strengths remaining relatively consistent, irrespective of layer heights, when tested in direction D3. The compactness of inter-layers, under the pressure applied by the print head to achieve layer heights smaller or equal to the nozzle diameter, which is the case in this study, has been shown in [54].

On the other hand, the compressive strengths of the 3D printed specimens show a ~20% reduction when tested in the D2 direction, when the layer height was increased to 20 mm, because of the much weaker inter-filament interface at higher layer heights. It needs to be noted that, as the layer heights increase, printing filaments using a circular nozzle result in curved edges, and thus, a higher layer height results in larger inter-filament defects, as shown in Figure 8. However, the use of rectangular nozzles has also been shown to result in anisotropic mechanical properties [23,35,43], but the extent of anisotropy as compared to those printed using circular nozzles, as reported, is rather scattered. It has also been noticed, from Figure 9, that the reduction in compressive strength in the D2 direction, with an increase in layer height, has been mitigated to a certain extend by the introduction of fibers. The fiber type and its orientation will influence the magnitude of efficiency in mitigating the effects of layer discontinuities [30,36,43,68], but it is seen here that, at higher layer heights, basalt fiber is more effective in mitigating strength loss. A plausible reason for this could be that some of the stiff steel fibers scratches the surface of the layers while printing, thereby causing surface defects. The lack of nozzle pressure at higher layer thickness is insufficient to overcome this effect.

Finally, as seen in Figure 4, in the D1 direction, both the inter-layer and inter-filament interfaces are parallel to the loading direction, and thus, they influence the failure under compressive loading. However, the number of inter-layer interfaces in the D1 direction is more than the number of inter-filament interfaces (see Table 5) when the print layer height is 6 mm or 13 mm. In this case, smaller layer heights result in the rounded filament edges causing smaller discontinuities, and thus, the presence of a larger number of inter-layer interfaces has a larger influence on failure. This is seen in Figure 9 where the compressive strength of the mixtures in the D1 direction is similar for 6 mm and 13 mm layer height specimens. An increase in layer height to 20 mm results in a similar number of inter-layer and inter-filament interfaces, as can be seen in Table 5. Larger inter-filament defects (see Figure 8) result in a significant reduction in the compressive strengths of these specimens compared to those printed with 6 mm and 13 mm layer heights. The reduction in strength in the D1 direction, when the layer height was increased from 6 mm to 20 mm, was ~15–20% for all the mixtures.

### 3.3. Anisotropy Quantification and Layer Size Effects

**Figure 10:**
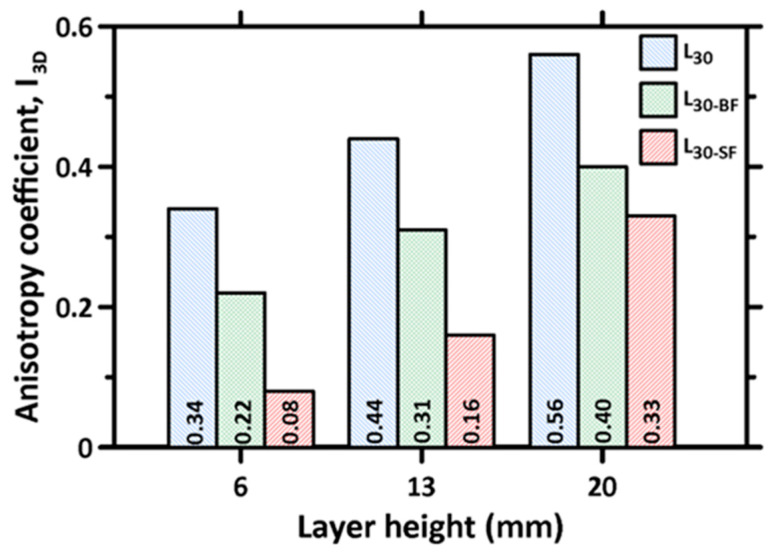
Calculated anisotropy parameters for the plain and fiber-reinforced mixtures corresponding to each layer height used in the study.

As mentioned earlier, the layer-by-layer stacking process in 3D printed concrete specimens is generally reported to result in an anisotropic response under mechanical loads [30,36,44,49]. The main factor that induces anisotropy in the 3D printed specimens is the difference in inter-layer and inter-filament interfacial properties, especially the porosity in this region. A dimensionless coefficient, I_3D_, given by Equation (1) [36,43], is used to characterize the anisotropy as:

(1) $$I_{3D}=\sqrt{{\left({CS}_{D1}-{CS}_{C}\right)}^{2}+{\left({CS}_{D2}-{CS}_{C}\right)}^{2}+{\left({CS}_{D3}-{CS}_{C}\right)}^{2}}/{CS}_{C}$$

where CS_C_ represents the compressive strength of the mold-cast specimens, and CS_D1_, CS_D2_, and CS_D3_ represent the compressive strengths of the 3D printed specimens in the D1, D2, and D3 directions, respectively. I_3D_ is equal to 0 for the mold-cast sample; thus, the smaller the coefficient, the weaker the anisotropy. The calculated anisotropy coefficients for the plain and fiber-reinforced mortar mixtures, corresponding to each layer height, are shown in Figure 10. It can be noticed that the anisotropy coefficient increases significantly with increases in layer height, demonstrating the influence of inter-layer and inter-filament effects on the compression response (note that increasing layer heights also increases the inter-filament defect fraction and size, as shown in Figure 8). It should be noted that, in two of the directions (Directions 1 and 3; see Figure 4), the stress path in compression is parallel to the inter-layers, which has been shown to result in a more significant strength reduction earlier in this paper. This results in a larger value of the numerator in the above equation and, consequently, a larger anisotropy coefficient. Furthermore, increasing the layer height results in weaker inter-filament interfaces, as was discussed earlier, which also leads to strength reduction, resulting in an increase in the anisotropy coefficient. The anisotropy coefficient decreases with the incorporation of fibers, with the steel fiber-reinforced mixtures showing the lowest anisotropy coefficients. At the lowest layer height, where the influence of inter-filament interfaces is minimal, the incorporation of steel fibers is seen to result in a near-isotropic behavior as far as uniaxial compression is concerned. Here, the strength reductions, when compared to the mold-cast samples that are caused by layer/filament defects, are overcome by the additional load-carrying capacity of the fibers. While there is some strength reduction for a cast specimen (as compared to unreinforced specimen) because of the loosening effect and wall effect [71], the strengths of the printed specimens with lower layer heights are closer to these values because the interfacial defects in printed elements result in a strength drop contribution of, roughly, the same magnitude as those of the defects in a mold-cast fiber-reinforced specimen. With increasing layer height, the efficiency of the interfacial defects—especially inter-filament defects—in reducing the strength attains higher proportions. A similar effect is seen for basalt fiber-reinforced specimens, albeit at a subdued level.

The aforementioned discussion reveals that the manifested anisotropy effects under compression are a function of the layer height and the mixture type (e.g., fiber type, and probably the volume fraction as well, though this was not investigated here). Near-isotropy is achieved using a smaller layer height and the use of steel fibers in this study, the reasons for which were explained earlier. The compromise here is that lower layer heights require increased printing time, thereby affecting the speed of construction and productivity. It also needs to be noted that anisotropic behavior may be acceptable when the printed elements are only compressed in a particular direction [43], thereby eliminating the need to obtain isotropy under all conditions. However, from an analysis and design standpoint, to be able to use traditional methodologies used for plain and reinforced concrete in structures that satisfy conventional functions, isotropy is a required criterion. It is also possible to beneficially use anisotropy when the predominant stress paths in a structure are known. This is the case when 3D printed topologically optimized structural members are used; this enables the optimized selection of printing paths and directions to obtain optimal performance, cost, and construction speed.

### 3.4. Stress–Strain Response in Compression

**Figure 11:**
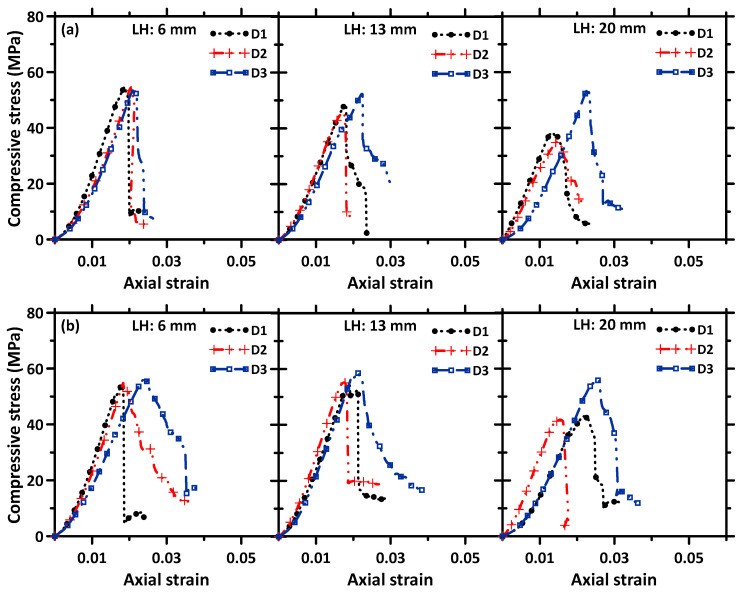
Representative stress–strain profiles extracted for (**a**) L30 and (**b**) L30-SF mortar specimens, for layer heights of 6 mm, 13 mm, and 20 mm, along the direction of printing (D1), layer-build-up direction (D2), and the direction (D3) perpendicular to both D1 and D2.

**Figure 12:**
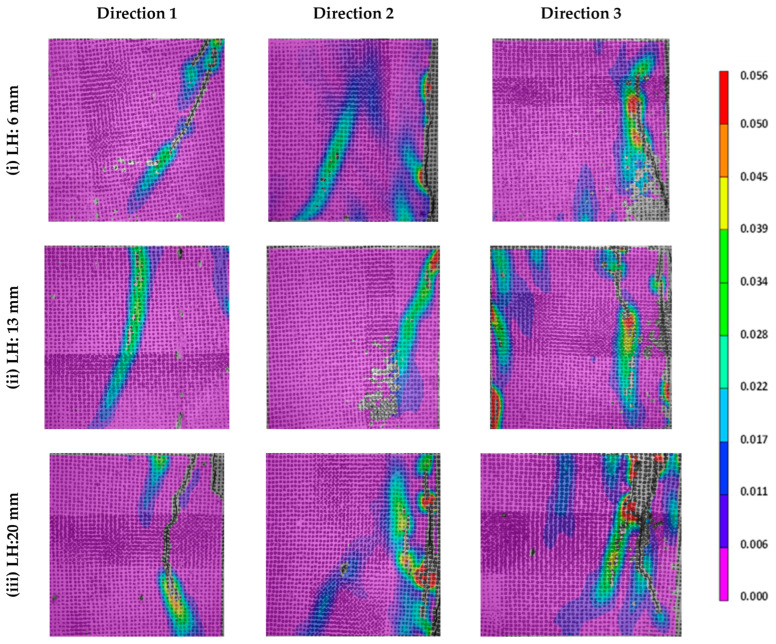
Principal strain profiles for L30-SF mortar specimens for the three layer heights and test directions used in study at peak stress.

The stress–strain responses of the 3D printed specimens in compression, with layer heights of 6 mm, 13 mm, and 20 mm and tested along the three mutually perpendicular directions, are shown in Figure 11 for the unreinforced and steel fiber-reinforced specimens. In the case of unreinforced specimens, the stress sharply drops once the peak load is reached when the layer height used was 6 mm, but a slight degree of post-peak softening is seen when the layer heights are higher. This could be attributed to the weaker inter-layer interfaces dissipating energy as the failure propagates through them. As a general observation, there is an enhancement in post-peak toughness in the D3 direction, as compared to the other two directions of testing—here, the stress path is perpendicular to the inter-filament interface, and the inter-layer interfaces dominate the failure. Observations of failure indicated that interface-parallel cracking and some crack branching happened in this case (see the DIC images in Figure 12 for the D3 direction, especially for the 20 mm layer height specimen, where the inter-filament interfaces are the weakest, as shown in Figure 8). The addition of fibers, in general, is seen to slightly enhance the compressive strength and the post-peak toughness, as compared to the unreinforced specimens, when tested in all three directions. As is expected, the addition of steel fibers is more beneficial in improving the toughness, but the beneficial effect of fibers is better demonstrated in tension and flexure, as opposed to compression, for reasons that are well-known.

**Figure 13:**
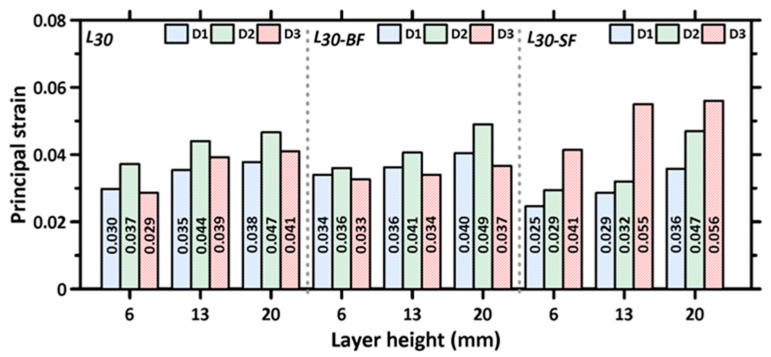
Maximum principal strain values for L30, L30-BF, and L30-SF mortar specimens, as a function of layer height, for each test direction used in the study at peak stress.

Typical DIC strain profiles at peak stress for the three tested directions and layer heights are shown in Figure 12 for the steel fiber-reinforced specimens. Only this set of specimens is shown here since the differences in principal strains, as a function of layer height, are much more discernible for these specimens than for unreinforced or basalt fiber-reinforced specimens. The strain profiles are similar for the other two cases as well, with the only changes being in the absolute magnitude of the strain values. The strain fields were determined from the displacement fields, which were evaluated using DIC [55]. The frames were extracted corresponding to the peak stress carried by the specimens under compression, with the Lagrangian principal strain as the contour variable. The principal strains are concentrated parallel to the direction of stress application, indicating that the interfaces parallel to the loading are more influential in the compressive response of the printed specimen (i.e., interface-parallel cracking and failure dominates, as described earlier), compared to those perpendicular to direction of applied stress. The maximum principal strain values at peak stress are obtained from DIC on the tested specimens for the 3D printed unreinforced and fiber-reinforced mixtures for the three layer heights, and they are shown in Figure 13.

The maximum principal strains are found to increase with an increase in layer height in all the three directions for all the specimens. The higher number of denser interfaces in the specimens printed using smaller layer heights is responsible for lower strains in those cases. For each layer height, the maximum principal strains are the highest when the loading is applied in the D2 direction for the unreinforced and basalt fiber-reinforced mixtures. Note that, when the loading is applied in the D2 direction, the inter-filament interfaces (which are the weaker interfaces) are parallel to the loading direction (see Figure 4), and the principal strain profiles are extracted on the surface, exposing the inter-filament interfaces to the DIC cameras. This is in line with the above discussions that brought out the dominant effects of inter-filament interfaces on the compressive strength, as well as the anisotropic nature of strength. This reinforces the need to choose the print layer heights judiciously to minimize voids at the inter-filament interfaces. For the specimens that were printed using steel fiber-reinforced mortar, the maximum principal strains are the highest in the D3 direction. This is demonstrated through multiple cracks, as noted in the DIC images (Figure 12, third column), especially at larger layer heights—an indication of the engagement of fibers in load transfer. The stiff steel fibers are generally oriented perpendicularly to the loading direction in this case (fibers generally orient in the print direction in 3D printed specimens, as shown in several studies including our previous work [54]), thereby resulting in an enhancement in post-peak toughness [72], which is also evident in the stress–strain responses shown in Figure 11. 

The principal strains for the unreinforced and fiber-reinforced mixtures at 60% of the peak stress were about 3–5 times lower than those at the peak stress, indicating that no significant crack propagation occurs until the specimens are loaded to ~60% of the peak stress, which is expected. However, at 80% of the peak stress value, the principal strains were only about 1.5 times lower than those at the peak stress, indicating significant crack development and/or separation of interfaces, which is further exacerbated close to the peak stress to result in complete failure.

**Figure 14:**
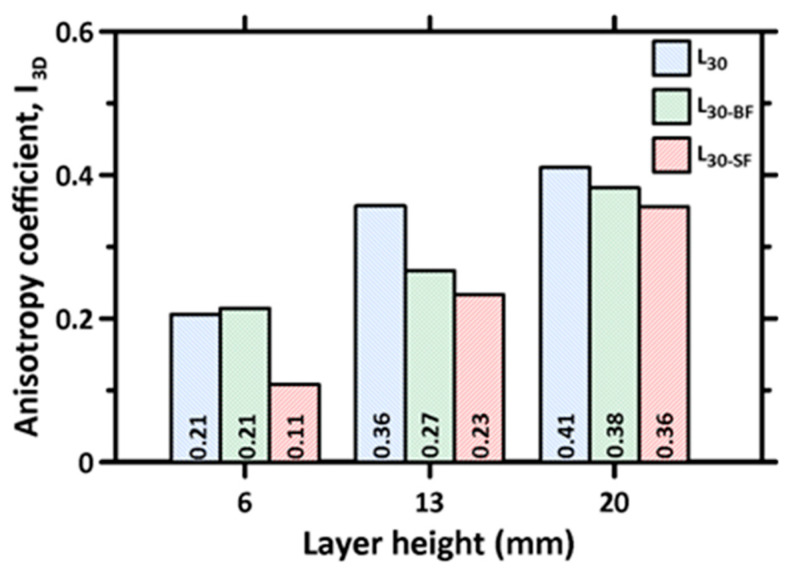
Principal strain-based anisotropy parameters for the plain and fiber-reinforced mixtures, corresponding to each layer height used in the study.

**Figure 15:**
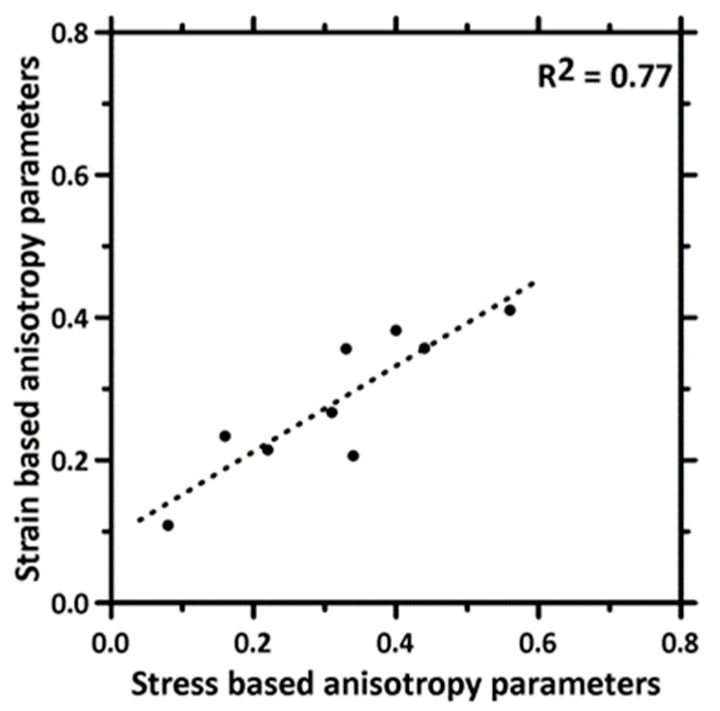
Correlation between anisotropy parameters obtained based on peak stresses and strains.

An anisotropy coefficient, similar to that discussed earlier, is calculated based on the peak principal strain values at the maximum stress using Equation (2):

(2) $$\varepsilon_{3D}=\sqrt{{\left(\varepsilon_{D1}-\varepsilon_{C}\right)}^{2}+{\left(\varepsilon_{D2}-\varepsilon_{C}\right)}^{2}+{\left(\varepsilon_{D3}-\varepsilon_{C}\right)}^{2}}\;/\;\varepsilon_{C}$$

where ε_C_ represents the maximum principal strain in the mold-cast specimen at the peak compressive stress (failure), and ε_D1_, ε_D2_, and ε_D3_ represent the maximum principal strains in the 3D printed specimens in the D1, D2, and D3 directions, respectively, at the peak stress. The calculated strain anisotropy coefficients for the plain and fiber-reinforced mortar mixtures, corresponding to each layer height, are shown in Figure 14. The anisotropy coefficients based on the strain values behave similarly to those based on the stress values but with overall smaller magnitudes. In other words, anisotropy in peak stress is more discernable for 3D printed specimens based on the results reported in this work. A reason for this observation is that, when a crack develops and propagates, the local strain in the region drops, and therefore, the strain differences in all the terms in the numerator of Equation (2) also reduce. However, it is easy to notice, from Figure 15, that the trend in strain anisotropy with layer heights is similar to that of stress anisotropy shown earlier. A linear relationship is also obtained between the stress-based and strain-based anisotropy parameters, even though the strain-based parameters have lower values.

## 4. Conclusions

This study has elucidated the influence of print layer height—and the consequent inter-layer and inter-filament interface properties—on the direction dependence of compressive strength and strains (anisotropy) of plain and fiber-reinforced (containing 0.28% by volume of basalt or chopped steel fibers) 3D printed mortar specimens. Slabs were 3D printed using three different layer heights corresponding to 0.30, 0.65, and 1 times the nozzle diameter (20 mm), and cubes extracted from them were tested in three orthogonal directions (denoted as D1, D2, and D3) to quantify the anisotropy in compressive stresses and strains, as well as to discern the mechanisms behind those same factors. The major findings of this study are:

- The direction-dependent compressive strengths of the 3D printed specimens were lower than those of conventionally cast samples for all layer heights considered; this observation was invariant of fiber type. The presence of interfacial defects (both inter-layer and inter-filament) reduced the compressive strengths in the case of 3D printed samples, which are layered systems, with an increase in layer height increasing the magnitude of strength reduction.
- The use of fiber reinforcement was observed to reduce the strength loss in 3D printed specimens compared to the conventionally cast mixtures, with the use of steel fiber and a lower layer height, resulting in the least strength disparity.
- The effect of print layer heights on the compressive strengths was also quantified for each test direction. The interfacial (inter-layer or inter-filament) contacts parallel to the stress direction were found to be more significant in compression, resulting in interface-parallel cracking to be the major failure mechanism. Thus, when the stress path was perpendicular to the weaker inter-filament interfaces, as in the case of direction D3, the strengths were less dependent on the layer height.
- In the D1 direction, both the inter-layer and inter-filament interfaces influenced the failure under compressive loading since they are both parallel to the load path, while in the D2 direction, the weaker inter-filament interfaces, alone, are parallel to the load path, thus exacerbating interface-parallel cracking. In direction D3, the crack paths faced interference by the inter-filament interfaces, which resulted in a somewhat notable post-peak toughness in the D3 direction as compared to the other two directions of testing, an observation which was corroborated by principal strains obtained from DIC as well. The maximum principal strains, determined using DIC, were also found to increase with layer heights in all the three directions.
- Using the directional compressive strengths and the principal strains at the peak stress (determined from DIC), stress and strain-based anisotropy coefficients were developed. The stress and strain-based anisotropy coefficients were found to be linearly related, even though the peak strain-based coefficients were slightly lower, because of the strain drop when cracking initiated. The lower the anisotropy coefficient, the lower the demonstrated anisotropy, as observed for specimens with a smaller layer height or when fibers (steel fibers in particular) were introduced to the mixture. The anisotropy coefficient increased with an increase in the print layer height, reflecting the influence of rather heterogeneous interfacial characteristics as the layer height increased.
- It has been conclusively shown that, by introducing fiber reinforcement in the mixture, the mechanical anisotropy in 3D printed specimens can be minimized. Stiff fibers such as steel fibers, when used appropriately, improve the mechanical response and reduce the anisotropy while ensuring that a higher print layer height can be chosen, yet reducing the weaker interfaces and, thereby, enhancing the speed of construction.
- Minimizing anisotropy is critical in ensuring that conventional design strategies (that consider the material to be homogeneous and isotropic) can be applied to 3D printed concrete elements as well.

## Figures and Tables

**Table 2:** Chemical composition and physical properties of the mortar components.

Components of the Binders	Chemical Composition (% by Mass)							d_50_ (μm)	Specific Gravity
	SiO_2_	Al_2_O_3_	Fe_2_O_3_	CaO	MgO	SO_3_	LOI *		
OPC	19.60	4.09	3.39	63.21	3.37	3.17	2.54	10.4	3.15
Limestone(L)	CaCO_3_ > 99%							1.5	2.70
Medium Sand (M)	SiO_2_ > 99%							200	2.40

* Loss on Ignition.

**Table 3:** Properties of the fibers used in the study.

Type of Fiber	Diameter (mm)	Length (mm)	Specific Gravity	Tensile Strength (GPa)	Young’s Modulus (GPa)
Chopped Basalt Fiber (BF)	0.04–0.06	15	2.36	0.28	18
Chopped Steel Fiber (SF)	0.20	13	6.8	3.0	210

**Table 4:** Mortar mixture proportions used in the study.

Mixture ID	Mass Fraction of Ingredients			Chopped Steel Fiber (SF) ^+^	Chopped Basalt Fiber (BF) ^+^	Water-to-Binder Ratio (w/b) by Mass	SP to Binder Ratio (SP%) by Mass of the Binder
	OPC	Limestone (L)	Sand (M)				
L30	0.35	0.15	0.5	-	-	0.35	0.35
L30-BF	0.35	0.15	0.5	-	0.28	0.35	0.35
L30-SF	0.35	0.15	0.5	0.28	-	0.35	0.35

^+^ Percentage by volume of the mixture.

**Table 5:** Number of inter-layer and inter-filament interfaces parallel to the loading (D1) direction for the 60 × 60 × 60 mm samples.

Layer Height	No. of Inter-Layer Interfaces	No. of Inter-Filament Interfaces
6 mm	8	2
13 mm	3	2
20 mm	2	2

## Data Availability

The raw/processed data required to reproduce these findings cannot be shared at this time as the data also forms part of an ongoing study.
